# Acinar cell carcinoma of gastric ectopic pancreas origin: a case report and literature review

**DOI:** 10.1186/s13000-023-01324-w

**Published:** 2023-03-16

**Authors:** Ying Chen, Ning Zhou, Deyu Guo, Xin He, Hao Tang, Lina Wang, Yujuan Xu, Tingting Xu

**Affiliations:** 1 Departments of Pathology, Guiqian International General Hospital, Guiyang, Guizhou Province, China; 2 Departments of Pathology, Sichuan Province, Sichuan Mianyang 404 Hospital, Mianyang, China

**Keywords:** Acinar cell carcinomas, Stomach, Ectopic, Pancreatic

## Abstract

**Background:**

Primary pancreatic-type acinar cell carcinoma of the stomach is extremely rare, often misdiagnosed, and of unclear origin.

**Case presentation:**

We report the case of a primary pure pancreatic-type acinar cell carcinoma of the stomach in a 58-year-old woman. This is the first reported case to exhibit residual ectopic pancreatic tissue adjacent to the tumor serving as evidence for the origin of the carcinoma. Furthermore, we summarized the clinicopathological features of pancreatic-type acinar cell carcinoma of the stomach in order to further understand this solid tumor.

**Conclusions:**

Primary pancreatic-type acinar cell carcinoma of the stomach is rare. Data on tumors of this histological type are still relatively scarce, and more in-depth research is needed to elucidate their molecular biological characteristics and prognosis.

## Background

Acinar cell carcinomas are relatively rare, accounting for 1–2% of all exocrine pancreatic tumors,while primary pancreatic-type acinar cell carcinomas of the stomach are even rarer, with only 9 cases (including the one presented here) reported in the literature over the past 20 years [1–8]. There has been much debate concerning the origin of this solid tumor. Here, we report a case of a 58-year-old female patient with primary pancreatic-type acinar cell carcinoma of the stomach. This was the first reported case to exhibit residual ectopic pancreatic tissue in the paracancerous region, indicating the origin of the tumor.

Notably, this tumor is prone to preoperative misdiagnosis, as all cases reported in the literature were diagnosed as tumors of other histological types based on preoperative pathological biopsy. By reviewing the past literature, this study aimed to summarize the clinicopathological characteristics of pancreatic-type acinar cell carcinoma of the stomach in order to further understand this solid tumor.

## Case presentation

**Fig. 1:**
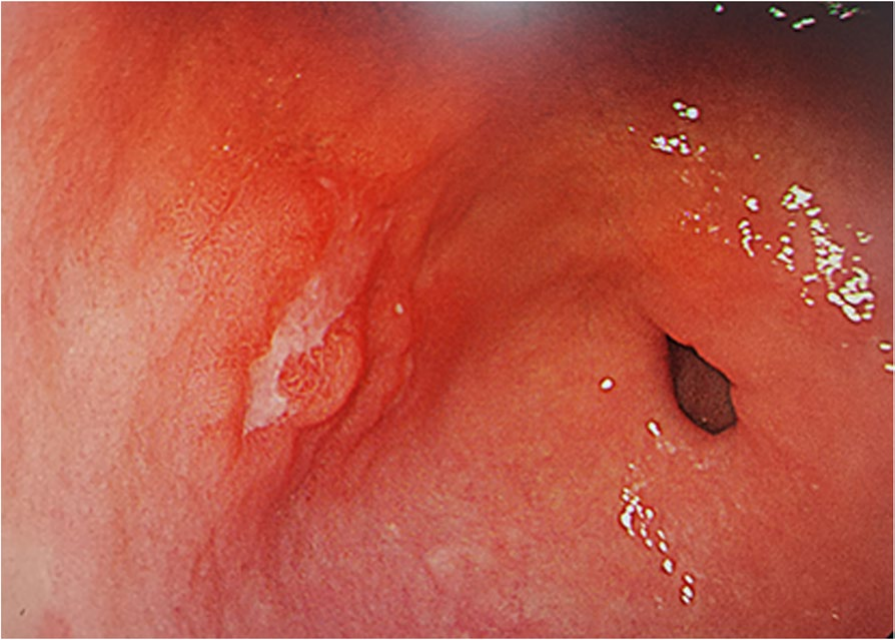
The electronic esophagogastroduodenoscopy performed at our hospital revealed a 1.8 cm × 1.5 cm lesion at the anterior wall of the gastric antrum, with ulcers on the surface

A 58-year-old female patient, who complained of upper abdominal pain and discomfort for more than 3 months, was diagnosed with gastric cancer through electronic gastroscopy at another hospital. The biopsy tissue showed poorly differentiated adenocarcinoma, and she was admitted to our hospital for further treatment and diagnosis. The electronic esophagogastroduodenoscopy performed at our hospital revealed a 1.8 cm × 1.5 cm lesion at the anterior wall of the gastric antrum, with ulcers on the surface (Fig. 1). Full abdominal computed tomography (CT) showed a slight thickening of the gastric wall at the antrum and no abnormalities in the liver, gallbladder, pancreas, spleen, and lymph nodes. Laparoscopic gastrectomy was performed after completing preoperative examinations.

**Fig. 2:**
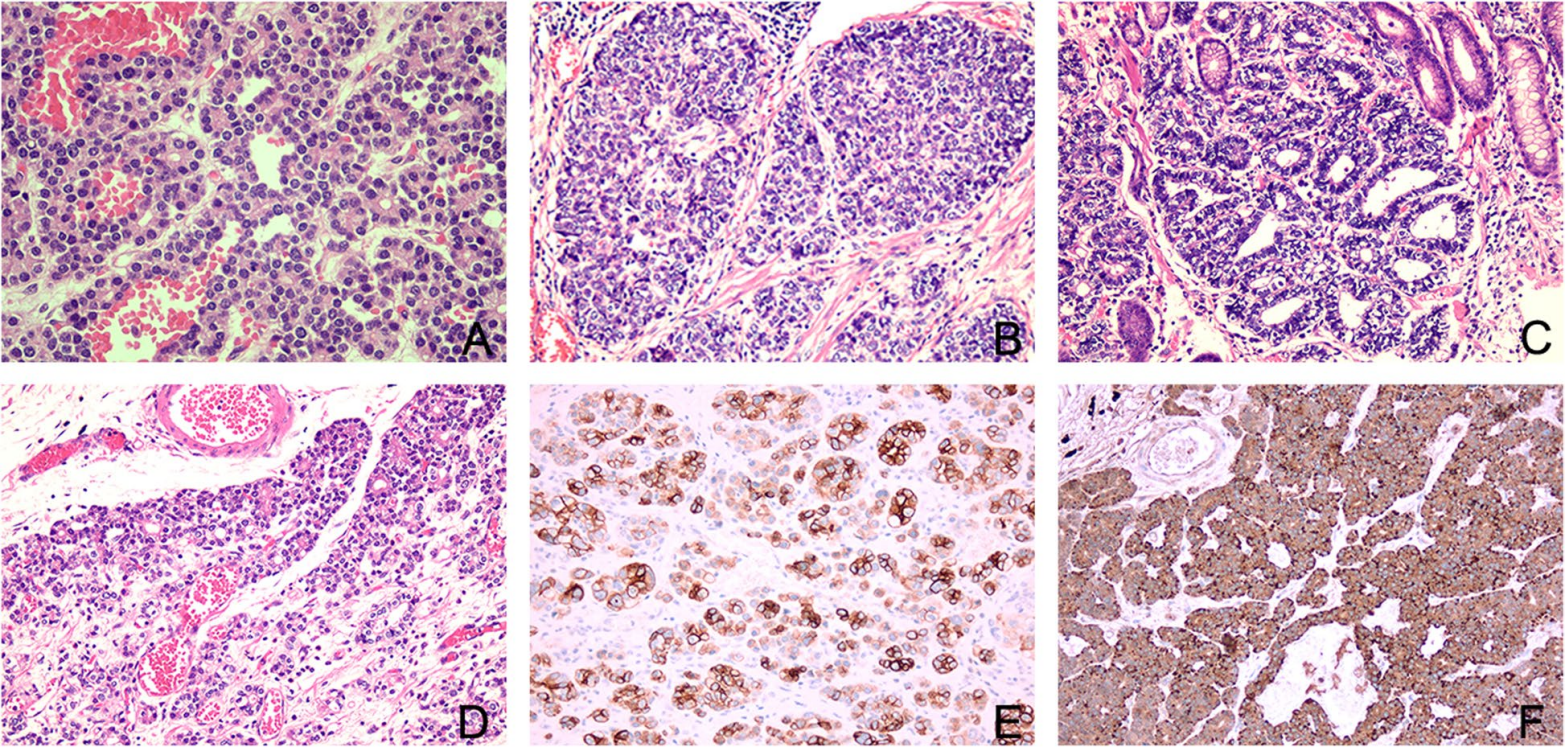
The tumor arranged in acinar pattern (**A** × 200). The tumor arranged in solid nest pattern (**B **× 200). The tumor arranged in glandular pattern (**C** × 200). Ectopic pancreatic tissue (**D** × 100). Immunohistochemical studies demonstrated diffuse positive staining for CK7 (**E** × 100), alpha-1-antitrypsin (**F** × 100)

Pathological examination revealed the gastric wall measuring 3.0 cm × 2.5 cm × 1.5 cm, with slightly exophytic erosions on the surface. The cut surface showed a poorly-circumscribed white area measuring 1.2 cm × 1.0 cm × 0.3 cm. The tumor was mainly located in the submucosa. The tumor was highly cellular, which grew in a lobular shape and invaded the muscularis mucosa; the mucosa was multifocally involved. The tumor cells were arranged in different architectural features, partially arranged in an acinar pattern (Fig. 2A). These cells had moderate amounts of granular eosinophilic cytoplasm containing zymogen granules, which were PAS-positive.Partially in a solid nest pattern,cells dysplasia were obvious (Fig. 2B). Some tumor cells were arranged in a glandular pattern (Fig. 2C).

Furthermore, we found ectopic pancreatic tissue in the adjacent tumor. It showed clear, sharp boundaries and fully developed acini and ductal structures (Fig. 2D); there were obvious transitions with the cancer tissue. The immunohistochemical workup was notable for CK19, CK7 (Fig. 2E), and alpha-1-antitrypsin (Fig. 2F), positivity in the tumor cells. The results of all tests performed with site-specific markers are presented (Table 1). The final histological and immunohistochemical results confirmed the diagnosis of primary pancreatic-type acinar cell carcinoma of the stomach. And the patient recovered well, with no recurrence or metastasis 6 months after surgery.

**Table 1:** Performed immunohistochemical stains, with interpretation and technical data

**Antibody**	**Result**	**Manufacturer**	**Species**	**Clone**	**Dilution**	**Stainer**
CK19	positive	MXB	mouse	MX054	predilute	ventana
CK7	positive	MXB	mouse	MX053	predilute	ventana
CK20	negative	MXB	mouse	MX059	predilute	ventana
CD56	Focal positive	MXB	mouse	MX039	predilute	ventana
CgA	Focal positive	MXB	mouse	MX018	predilute	ventana
SYN	negative	MXB	mouse	MX038	predilute	ventana
alpha-1-antitrypsin	positive	MXB	rabbit	/	predilute	ventana
Ki67	Index 40%	MXB	rabbit	MXR002	predilute	ventana

*Abbreviation: MXB* Maxim Biotech Co

## Discussion and conclusions

Primary pure pancreatic-type acinar cell carcinomas of the stomach are relatively rare, with only 9 cases (including the one presented here) reported in the literature over the past 20 years. The 9 cases (Table 2) included 4 females and 5 males, with a median age of 63 years and no significant difference in sex ratio. The tumor was located at the gastric antrum in 4 cases, at the gastric fundus in 2 cases, and at the gastric body, cardia, and pylorus, respectively, in the remaining 3 cases.

**Table 2:** Reported cases of primary pancreatic-type acinar cell carcinoma of the stomach

Case	Author & Year	Sex	Age (year)	Pre-op diagnosis	Site	Gross morphology	Maximum tumor diameter (cm)	Pancreatic metaplasia/ ectopic pancreas	Surgical procedure	Metastasis	Follow-up
1	Sun et al. 2004 [1]	F	86	Poorly differentiated adenocarcinoma	Antrum	Exophytic polypoid lesion with ulceration	5.0	None	Partial gastrectomy	NM	NM
2	Mizuno et al. 2007 [2]	M	73	Gastrointestinal stromal tumor	Pylorus	Polypoid exophytic lesion	7.6	None	Pancreaticoduodenectomy	Lymph nodes, liver	Survived 11 months
3	Ambrosini-Spaltro et al. 2009 [3]	M	52	Poorly differentiated adenocarcinoma	Antrum	Ulcerated exophytic lesion	4.0	Pancreatic metaplasia	Subtotal gastrectomy	None	NM
4	Coyne [4]	F	77	Poorly differentiated adenocarcinoma	Fundus	Lobulated exophytic lesion	4.5	None	Partial gastrectomy	None	Died after 1 month
5	Yonenaga et al. [5]	M	63	Poorly differentiated adenocarcinoma	Antrum	Ulcerated exophytic lesion	6.5	None	Partial gastrectomy	Lymph nodes and liver	Died after 5 months
6	Kim et al. [6]	M	54	Gastrointestinal stromal tumor or lymphoma	Cardia	Polypoid exophytic lesion	2.7	None	Partial gastrectomy	None	Survived 33 months
7	Uno et al. [7]	M	68	Poorly differentiated adenocarcinoma	Body	Depressed lesion	1.7	None	ESD	None	NM
8	Paseiro-Crespo et al. [8]	F	51	high-grade neuroendocrine tumor	Fundus	Lobulated exophytic lesion	8	None	Total gastrectomy	Liver	Survived 5 months
9	This case	F	58	Poorly differentiated adenocarcinoma	Antrum	Ulcerated exophytic lesion	1.2	Ectopic pancreas	Partial gastrectomy	None	Survived 6 months

*Abbreviation*:*F* Female, *M* male, *NM* Not mention

There are currently three hypotheses concerning the origin of extra-pancreatic acinar cell carcinoma [8]. The first hypothesis proposes that the tumor originates from the pancreatic metaplasia of the gastric mucosal epithelium. Pancreatic metaplasia is most commonly found in the cardia mucosa and frequently occurs within the context of autoimmune gastritis [9]. Among the 9 cases above, only one [3] clearly exhibited pancreatic metaplasia of non-neoplastic gastric mucosa, whereas the tumors in all the cases occurred submucosally, making it difficult to explain their origin using metaplasia.

In the second hypothesis, the tumor is thought to originate from pluripotent stem cells with different directions of differentiation [10]. However, most researchers currently favor the third hypothesis, which proposes that the tumor originates from the ectopic pancreas. The stomach and pancreas are both derived from the caudal or distal part of the embryonic foregut, and the abnormal differentiation of their inherent stem cells may be the origin of ectopic pancreases. According to relevant literature, the incidence of ectopic pancreas is 2%–15% [11]. Although ectopic pancreas can occur throughout the entire gastrointestinal tract, it is most commonly found in the stomach. The ectopic pancreas in the stomach is mainly under the gastric antrum mucosa, which is consistent with most tumor sites reported in the literature. Ectopic pancreas can become malignant, with a malignant transformation rate of 0.7%–1.8% [11]. However, acinar cell carcinoma of ectopic pancreas origin is still controversial because ectopic pancreatic tissue has not been found in the previously reported literature. Our patient had the typical histological structure and immunophenotype of acinar cell carcinoma. For the first time, we found residual ectopic pancreatic tissue in the paracancerous region. It showed clear and sharp boundaries and fully developed acini and ductal structures. The transition between pancreatic structures and carcinoma could be observed, indicating the origin of the tumor.

After reviewing the literature, we also found a problem worthy of attention. The preoperative misdiagnosis and missed diagnosis rate of gastric pancreatic acinar cell carcinoma are as high as 100%. Among the existing cases, 6 (including the present one) were diagnosed preoperatively as poorly differentiated adenocarcinoma, 1 was diagnosed as a high-grade neuroendocrine tumor, whereas no tumors were detected in the gastric mucosa of the remaining 2 cases. The reasons for missed diagnosis and misdiagnosis may include the following: (1) Although the tumors mainly presented as polypoid exophytic lesions with ulceration, the main body of the lesion was located submucosally, which meant that mucosal biopsies were prone to a missed diagnosis. (2) Cases with mucosal invasion by the tumor (especially the solid and the glandular types) were easily misdiagnosed as poorly differentiated adenocarcinoma [7]. In addition, 1 case reported in the literature was misdiagnosed as a high-grade neuroendocrine tumor due to their morphological similarity and the expression of neuroendocrine markers in acinar cell carcinoma. In fact, 42% of acinar cell carcinomas have been shown to express neuroendocrine markers [12], but mostly exhibited focal positives. Thus, special attention should be paid to the presence of acinar differentiation to prevent misdiagnosis, and IHC testing should be performed. Trypsin, chymotrypsin, a-1-Antitrypsin, and BCL10 antibodies are the most sensitive. The overall prognosis for acinar cell carcinoma is relatively poor, with median survival age of 22 months and a 5-year survival rate of 21.5% [13]. However, it is unclear whether primary pancreatic-type acinar cell carcinoma of the stomach has a similar prognosis. Among the 9 cases reviewed in this study, 5 underwent partial gastrectomy, 3 underwent total gastrectomy, and 1 underwent endoscopic submucosal dissection (ESD). Among the 6 patients followed up, 4 developed metastasis and 2 died; the shortest survival time was 1 month. Taken together, these data suggest that the overall prognosis of extra-pancreatic acinar cell carcinoma is also relatively poor, and the risk factors for poor prognosis may include tumor size and the presence of metastasis.

In conclusion, primary pancreatic-type acinar cell carcinoma of the stomach is rare. This case was the first in which a residual ectopic pancreas was found in the paracancerous region, indicating the origin of the tumor. Data on tumors of this histological type are still relatively scarce, and more in-depth research is needed to elucidate their clinicopathological features.
